# Health Literacy predicts incident foot ulcers after 4 years – the SHELLED cohort study

**DOI:** 10.1186/s13047-023-00644-w

**Published:** 2023-07-27

**Authors:** Pamela Chen, Michele Callisaya, Karen Wills, Timothy Greenaway, Tania Winzenberg

**Affiliations:** 1 School of Medicine, Faculty of Health, University of Tasmania, 17 Liverpool Street, Hobart, TAS Australia; 2 Present Address: Joondalup Health Campus, Ramsay Healthcare Australia, Corner Shenton Ave and Grand Boulevard, Joondalup, WA 6027 Australia; 3 Menzies Institute for Medical Research, University of Tasmania, 17 Liverpool Street, Hobart, TAS Australia

**Keywords:** Cognition, Complications, Diabetic foot, Health literacy, Prevention

## Abstract

**Aims/hypothesis:**

To determine whether health literacy is associated with an index diabetes-related foot ulcer (DFU).

**Methods:**

The SHELLED Study is a 4-year prospective study of people with diabetes aged over 40 with no history of DFU. The primary outcome was development of a first foot ulcer. Health Literacy was measured using the short form Test of Functional Health Literacy in Adults (s-TOFHLA) and nine domains of the Health Literacy Questionnaire (HLQ).

**Results:**

Of 222 participants, 191 (86.0%) completed the study, of whom 13 (5.9%) developed an incident ulcer. In multivariable models, every unit increase in S-TOFHLA was associated with a reduced odds of foot ulcer development by 6% (OR 0.94, 95% CI 0.88 to 0.99). Better scores on two HLQ domains reduced the odds of foot ulcer (*actively managing my health* (OR 0.23, 95% CI 0.08 to 0.65) and *understanding health information well enough to know what to do* (OR 0.39, 95% CI 0.19 to 0.78). This was independent of baseline risk for foot disease.

**Conclusions/interpretation:**

These data provide novel evidence that health literacy is an important clinical risk factor for index foot ulceration. This is an area of potential focus for research and development of educational programs or policy aimed at reducing development of incident foot ulceration.

**Supplementary Information:**

The online version contains supplementary material available at 10.1186/s13047-023-00644-w.

## Introduction

Diabetes-related foot disease is one of the most devastating end-stage complications of diabetes. It is a leading cause of diabetes related disability burden [1] and affects up to 26.1 million people worldwide annually [2]. It precedes up to 75% of amputations in people with diabetes [3], and has unacceptably high mortality rates. The 5-year life expectancy of someone with diabetes-related foot disease is around 40% [4]. In addition, diabetes-related foot disease is detrimental to mental and emotional health [5], and is the leading cause of diabetes-related hospitalizations globally [3].

Identification of people at risk and taking appropriate preventative interventions as part of integrated foot care are considered cornerstones to preventing index or recurrent diabetes-related foot ulcers [6, 7]. These include professional foot care, structured education, preventative footwear and regular foot examinations [8]. However, on top of maintaining optimal glycemic control through glucose management and lifestyle changes, these components of behavioural change can be complex and overwhelming for patients. Interactions between healthcare provider and consumer, and ability to incorporate education or counselling on effective behaviour change may be key to improving ulcer prevention as part of wider biopsychosocial models of care [9].

A critical component underpinning the interactions between healthcare consumers and providers and the wider health system is health literacy. One way of defining health literacy is as ‘the cognitive and social skills which determine the motivation and ability of individuals to gain access to, understand, and use information in ways which promote and maintain good health’ [10]. However, the definition of health literacy continues to evolve as a new construct, and has had numerous definitions based on varying interpretations of it as an individual skill or at a societal level [11]. The schema proposed by Nutbeam [10] of health literacy as an individual skill describes the most basic functional health literacy as reading or writing skills required to be able to understand health information. Communicative or interactive health literacy is more complex and involves skills to extract and derive meaning from different types of communication. The most advanced critical health literacy requires skills in analysis of information and applying this to personal health circumstances [10]. These are arguably crucial skills required to successfully manage diabetes, as well as to adhere to recommendations needed to prevent foot ulceration [12, 13].

Health literacy deficits are a major barrier to self-care in people with diabetes [14]. An individual with poor health literacy, or whose health literacy needs are inadequately supported, can be disadvantaged when attempting to engage in strategies for diabetes management and foot ulcer prevention [15]. There are established associations between poor health literacy and diabetes-related complications of retinopathy and cerebrovascular disease [16], and limited cross-sectional data suggesting functional health literacy may be associated with foot ulceration [17]. Whilst we have previously reported no relationship between functional health literacy and risk factors for foot disease from cross-sectional data from this study [18], the importance of other aspects of health literacy, as well as longitudinal data which would strengthen the cross-sectional evidence, is lacking. Therefore, the aim of this study was to examine the association between health literacy and the development of an index diabetes-related foot ulcer over 4 years. Understanding this key relationship may facilitate improvement of diabetes-related foot disease prevention.

## Subjects

The Southern Tasmanian Health Literacy and Foot Ulcer Development in Diabetes (SHELLED) study is a 4-year longitudinal study aiming to determine the associations of health literacy with incident foot ulceration in people with diabetes.

Participants were recruited from the Royal Hobart Hospital’s Diabetes Centre Outpatient clinics between January 2015 and July 2016. Details on recruitment, data collection and questionnaires used have been described elsewhere [18]. In brief, participants were eligible if they were aged > 40 and had established diabetes mellitus diagnosed according to WHO criteria [19]. Exclusion criteria included those with a history of amputation, ulceration, a diagnosis of peripheral neuropathy attributed to causes other than diabetes, psychotic disorders, dementia, blindness, or were unable to converse in English.

## Materials and methods

This was a 4-year prospective longitudinal study aimed at determining whether health literacy predicted occurrence of an index diabetes-related foot ulcer. The primary outcome was whether a participant developed an index diabetes-related foot ulcer (defined as a full thickness lesion below the ankle that was present for > 2 weeks) during the 4-year follow-up period. This was assessed by self-report during annual phone follow-ups conducted by a research assistant, with confirmation either via hospital medical records or by contacting the participant’s general practitioner.

The exposure of interest, health literacy was measured using the short form Test of Functional Health Literacy in Adults (S-TOFHLA) and the Health Literacy Questionnaire (HLQ). The S-TOFHLA is a timed, 36-item test of comprehension using a modified cloze procedure. Participants are required to complete two passages, one containing instructions to have an x-ray and the other, from the “patients’ rights and responsibilities” section of an American Medicaid application form [20]. The Australian equivalents of American terms were provided to participants prior to the test being administered. The S-TOFHLA has a scoring range of 0–36 and regularly categorized into adequate (> 22/36), marginal (17–22/36) and inadequate (< 17/36) functional health literacy respectively, although the cut-offs are population specific [21]. The S-TOFHLA is widely used and has excellent reliability (Cronbach’s alpha 0.98) and validity (0.91) [20, 22].

The HLQ is a more holistic assessment of health literacy, consisting of 9 scales assessing the following domains: 1. Feeling understood and supported by healthcare professionals (4 items); 2. Having sufficient information to manage my health (4 items); 3. Actively managing my health (5 items); 4. Social support for health (5 items); 5. Appraisal of health information (5 items); 6. Ability to actively engage with healthcare providers (5 items); 7. Navigating the health system (5 items); 8. Ability to find good health information (5 items); and 9. Understanding health information well enough to know what to do (5 items). Scales 1 to 5 are scored out of 4 (strongly disagree, disagree, agree, strongly agree). Scales 6 to 9 measure difficulty of health-related tasks by the individual, and are scored out of 5 (cannot do or always difficult, usually difficult, sometimes difficult, usually easy and always easy). Composite reliability of the HLQ ranges between 0.77 and 0.89 [23].

Risk factors for diabetes-related foot disease were assessed by a podiatrist according to the most current Australian guidelines [24] at the time the study protocol was developed (2014) and have been previously described [18]. In brief, they included testing for loss of protective sensation (using the Semmes-Weinstein 10 g monofilament and vibration perception), assessment of peripheral artery disease (Ankle-Brachial Index) and foot deformity using the 6-point foot deformity score. As per Australian National guidelines [24], participants were classified according to the number of these risk factors they had at the time of assessment into categories of low (0 risk factors), medium (1 risk factor) or high (2 or more risk factors) risk for diabetes-related foot disease.

Demographic characteristics (age, gender, employment status), medical history (duration and type of diabetes, insulin therapy), years of educational attainment and household income bracket were assessed by questionnaire. Other covariates identified as potentially influencing health literacy as well as foot ulcer development according to biopsychosocial models of care were also assessed included diabetes self-efficacy (Diabetes Management Self-Efficacy Scale [25]), foot care self-efficacy (Foot Care Confidence Scale [26]), depression (Patient Health Questionnaire-9 [27]), diabetes-related distress (Diabetes Distress Scale [28]), diabetes knowledge (Diabetes Knowledge Questionnaire [29]) and foot care behaviour (Foot Care Behaviour Scale) which were assessed by validated questionnaires as has previously been described [18]. The Montreal Cognitive Assessment (MOCA) [30]) was administered when participants attended for their foot assessment. The MOCA is a validated screening tool with scores < 26/30 indicative of mild cognitive impairment in people with diabetes [30].

### Statistics

The sample size of 220 was calculated as the number of participants needed to detect differences in associations of S-TOFHLA categories (adequate vs inadequate health literacy) with foot ulcer incidence over 4 years. We projected that 60% of our study sample would have inadequate health literacy based on estimates by the Australian Bureau of Statistics [31]. Based on global foot ulceration incidence of between 2 and 5% in developed countries [32, 33], we would have power at 80% to detect a 3.8% difference in foot ulcer incidence in people with inadequate and adequate health literacy.

Participants were considered lost to follow up if they were unable to be contacted at the completion of the 4^th^ year of follow up and had not had an incident ulcer, or if they were noted as deceased on their hospital medical records without a known incident ulcer. Those with incident ulceration who died before the end of follow-up were considered to have completed the study as they had attained the outcome of interest.

Logistic regression was used to estimate the associations of health literacy with development of incident foot ulceration over 4 years. Ten models were performed, one for S-TOFHLA scores and one for each of the nine health literacy domains measured by the HLQ [23]. Models were weighted according to the inverse probability of each participant not completing the 4 year follow up period. Odds Ratios indicate the odds of an individual developing an index foot ulcer with increasing scores for each independent variable. Potential confounders were selected based on clinical and biological plausibility and if considered not to be on the causal pathway between health literacy and foot ulcer development. Final models were selected based on residual deviance of overall models, and adequacy confirmed using the Hosmer-Lemeshow goodness of fit test.

All analyses were performed in R V 1.10.44 (R Core Team, 2018) using the package VGAM [34].

### Ethics

This study was approved by the University of Tasmania Human Research Ethics Committee (H0014284).

### Patient and public involvement

No patients were involved in setting the research question or the outcome measures, nor were they involved in developing plans for recruitment, design, or implementation of the study. No patients were asked to advise on interpretation or writing up of results. All participants were provided education on foot ulcer prevention and where concerns of depression (PHQ-9) were identified these were communicated to participants’ treating medical practitioner for follow up.

## Results

### Participant characteristics

**Fig. 1:**
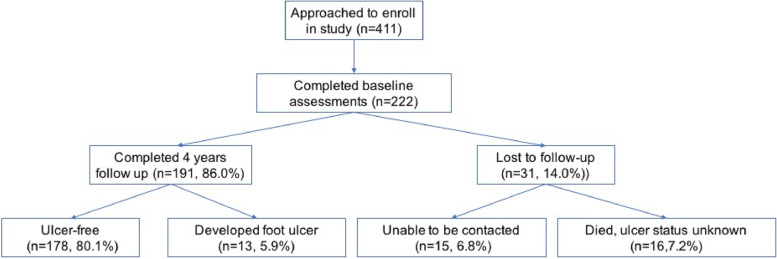
Participant flow chart

Figure 1 shows participant flow through the study. Of four hundred and eleven people who were approached, 222 enrolled and completed baseline assessments. At the end of 4 years, 191(86.0%) completed the study, of whom 178 (80.1%) were ulcer-free and 13 (5.9%) developed an incident ulcer. There were 25 deaths with a mortality rate of 11.3%. The 31 participants who were lost to follow up were older, more likely to be male, have a lower S-TOFHLA score, and had a longer duration of diabetes compared to those who completed the study (Supplementary Table 1).

Table 1 shows the baseline characteristics of the whole study sample and of participants by ulcer status at 4 years. Participants were predominantly (58.6%) male with mean age 60.5 (SD 10.7) years. Their mean duration of diabetes was 18.0 (SD 13.4) years and 173 (77.9%) were insulin dependent. The mean MOCA (cognition) score was 25.7 (SD 3.5) out of 30 and mean score on the Patient Health Questionnaire (PHQ-9) was 7.2 (SD 6.3). 127 (57%) were at low, 81 (36.5%) medium and 14 (6.3%) at high risk of foot disease at baseline [24]. Those who developed an incident ulcer had a higher BMI, lower S-TOFHLA, HLQ and MOCA scores, and were more likely to score higher on the PHQ-9 questionnaire than those who did not develop (Table 1) an incident ulcer.

**Table 1:** Baseline characteristics of the study sample and of participants by foot ulcer status after 4 years

**Variable**	**Whole sample (*n* = 222)**^**a**^	**Completed study (*n* = 191)**	
		Developed ulcer (*n* = 13)	Did not develop ulcer (*n* = 178)
**Age**	60.5 (10.7)	63.9 (12.1)	60.3 (10.1)
**Male, n(%)**	130 (58.6)	8 (61.7)	100 (56.2)
**Years of formal education**	11.3 (3.3)	10.1 (1.8)	11.5 (3.5)
**STOFHLA score (0–36)**	31.9 (6.7)	28.4 (10.2)	32.8 (5.2)
**HLQ Domain Scores**			
**1: Feeling understood and supported by health professionals**	3.26 (0.52)	3.11 (0.55)	3.26 (0.53)
**2: Having sufficient information to manage health**	3.06 (0.45)	2.96 (0.32)	3.07 (0.46)
**3. Actively managing my health**	2.81 (0.50)	2.40 (0.66)	2.83 (0.48)
**4. Social support for health**	2.97 (0.60)	2.75 (0.55)	2.96 (0.61)
**5. Appraisal of health information**	2.83 (0.57)	2.52 (0.67)	2.85 (0.56)
**6. Ability to actively engage with health professionals**	4.07 (0.69)	2.68 (1.03)	4.09 (0.67)
**7. Navigating the healthcare system**	3.94 (0.64)	3.63 (0.65)	3.96 (0.63)
**8. Ability to find good health information**	3.84 (0.76)	3.37 (0.89)	3.88 (0.75)
**9. Understanding health information well enough to know what to do**	4.00 (0.68)	3.42 (0.87)	4.05 (0.66)
**BMI (kg/m**^**2**^**)**	33.6 (8.1)	35.1 (11.8)	33.7 (8.0)
**Duration of diabetes (years)**	18.0 (13.4)	18.6 (9.9)	17.2 (13.6)
**Insulin therapy, n(%)**	173 (77.9)	12 (92.3)	135 (75.8)
**PHQ-9 (0–27)**	7.2 (6.3)	10.1 (7.7)	7.0 (6.2)
**Diabetes Distress (0–6)**	1.7 (0.8)	2.0 (0.9)	1.7 (0.8)
**DMSES (0–10)**	9.5 (1.7)	7.98 (1.78)	9.61 (1.74)
**MOCA (0–30)**	25.7 (3.5)	22.4 (4.3)	26.2 (3.2)
**Diabetes Knowledge (0–100)**	73.2 (19.0)	65.0 (24.2)	74.3 (18.1)
**Current smoker, n(%)**	33 (14.9)	1 (7.6)	29 (16.3)

Data presented as mean(SD) unless otherwise indicated. Numbers in brackets after each variable is the possible score range where relevant

*S-TOFHLA* Short form Test of Functional Health Literacy in Adults, *HLQ* Health Literacy Questionnaire, *BMI* Body Mass Index, *PHQ-9* Patient Health Questionnaire – 9 items, *DMSES* Diabetes Management Self-Efficacy Scale, *MOCA* Montreal Cognitive Assessment; HLQ domains 1–5 are scored out of 4, and domains 6–9 are scored out of 5

^a^Except for BMI, PHQ score and diabetes distress (*n* = 221) and years of formal education (*n* = 220)

### Associations of health literacy with incident foot ulcer development at 4 years

Table 2 shows the odds ratios (ORs) for development of an incident ulcer at 4 years per unit increase in health literacy in separate models for the S-TOFHLA and for each health literacy domain measured by the HLQ.

**Table 2:** Univariable and multivariable associations of each health literacy measure with odds of incident diabetes-related foot ulcer development over 4 years

**Variable**	**Univariable**		**Multivariable model**^**a**^		**Multivariable model**^**a**^** adjusted for cognition**	
	OR	95% CI	OR	95% CI	OR	95% CI
**S-TOFHLA model**						
**S-TOFHLA**	**0.92**	**0.86, 0.99**	**0.94**	**0.88, 0.99**	1.02	0.94, 1.12
**Models of HLQ domains**						
**Domain 1: Feeling understood and supported by health professionals**						
**HLQ – domain 1**	0.60	0.21, 1.72	0.53	0.19, 1.45	0.58	0.20, 1.62
**Domain 2: Having sufficient information to manage health**						
**HLQ – domain 2**	0.58	0.15, 2.05	0.59	0.16, 2.05	0.51	0.13, 1.88
**Domain 3: Actively managing my health**						
**HLQ – domain 3**	**0.20**	**0.06, 0.60**	**0.23**	**0.08, 0.65**	**0.17**	**0.05, 0.50**
**Domain 4: Social support for health**						
**HLQ – domain 4**	0.58	0.25, 1.39	0.53	0.24, 1.22	0.46	0.20, 1.12
**Domain 5: Appraisal of health information**						
**HLQ – domain 5**	0.40	0.16, 1.01	0.42	0.17, 1.04	0.47	0.19, 1.09
**Domain 6: Ability to actively engage with healthcare professionals**						
**HLQ – domain 6**	0.48	0.23, 1.005	0.56	0.28, 1.15	0.68	0.34, 1.36
**Domain 7: Navigating the healthcare system**						
**HLQ – domain 7**	0.49	0.22, 1.11	0.56	0.26, 1.24	0.63	0.29, 1.35
**Domain 8: Ability to find good health information**						
**HLQ – domain 8**	**0.48**	**0.25, 0.93**	0.56	0.30, 1.06	0.72	0.39, 1.33
**Domain 9: Understanding health information well enough to know what to do**						
**HLQ – domain 9**	**0.32**	**0.15, 0.67**	**0.39**	**0.19, 0.78**	0.55	0.27, 1.10

Bold denotes statistically significant

Models weighted according to inverse probability of each participant remaining in the study over 4 years

*Abbreviations*: *S-TOFHLA* Short form Test of Functional Health Literacy in Adult, *MOCA* Montreal Cognitive Assessment, *HLQ* Health Literacy Questionnaire

^a^Multivariable models adjusted for years of formal education, age, gender and BMI

After adjustment for age, gender, BMI and education level, better health literacy scores on the S-TOFHLA and two HLQ domains were protective against foot ulcer development. Every unit increase in S-TOFHLA score reduced the odds of foot ulcer development by 6% (OR 0.94, 95% CI 0.88, 0.99) (Table 2). Of the HLQ domains, *actively managing my health* and *understanding health information well enough to know what to do* were also associated with a first foot ulcer. For the former, each unit increase in mean HLQ domain score (i.e. a 1-unit improvement on the 4-point Likert scale) was associated with a 77% reduction in odds of incident foot ulcer (OR 0.23, 95% CI 0.08, 0.65). For the latter, each unit increase (i.e. a 1-unit improvement on the 5-point Likert scale) was associated with a 61% reduction in odds of a first foot ulcer (OR 0.39, 95%CI 0.19, 0.78).

After further adjustment of the models for cognition (MOCA score), only the protective effect of the HLQ domain *actively managing my health* (OR 0.17, 95% CI 0.05, 0.50) persisted. Scores on the MOCA were statistically significantly associated with index foot ulceration in all models, with reductions in odds ratios ranging for 17–23% per unit increase in MOCA score. Odds ratios ranged from 0.83 (95%CI (0.72 to 0.96) for domain 9, to 0.77 (95%CI 0.65 to 0.88) and 0.77 (95%CI 0.63 to 0.94) for domain 3 and the S-TOFHLA respectively. Further adjustment for duration of diabetes, insulin therapy, baseline category of risk for foot disease at study enrolment, diabetes distress and depression or any other covariate measured did not materially change the magnitude or statistical inference of results (data not shown).

## Discussion

This longitudinal study provides novel data that functional and multiple domains of health literacy may be crucial in preventing a first foot ulcer. It is the first to prospectively investigate the relationship between health literacy and incident foot ulcer development in people with diabetes, enhancing previous evidence from cross-sectional data on this relationship [17]. Two domains of the HLQ, *understanding health information well enough to know what to do* and *actively managing my health* as well as functional health literacy measured by the S-TOFHLA were associated with potentially clinically important reductions of up to 77% in the odds of developing a foot ulcer at 4 years. Additionally, cognitive impairment was independently associated with foot ulceration in all models, with every unit increase in MOCA score reducing the odds of incident foot ulcer development by between 17 and 23%. Consideration should be given as to whether policymakers and health care providers should identify people with health literacy and cognitive deficits so as to target them for interventions to improve health literacy, and tailor educational programs and other interventions for diabetes-related foot disease prevention to meet their needs.

Health literacy could have a key role to play in incident foot ulcer prevention. In this study, both S-TOFHLA and HLQ domain of *understanding health information well enough to know what to do* performed similarly in regression models and better scores were protective for incident foot ulceration. This is unsurprising. The HLQ domain *understanding health information well enough to know what to do* broadly matches the Nutbeam schema of functional health literacy [23] so individuals scoring poorly on this domain are expected to have problems understanding written health information or instructions about treatment or medications, and are unable to read or write well enough to complete medical forms [23]. By extension, they would thus be expected to perform poorly on the S-TOFHLA, which tests these capabilities [20]. Evidence from previous cross-sectional data from two studies of 1278 participants pooled by meta-analysis demonstrated a clinically important, but not statistically significant doubling of the odds of foot disease among people with inadequate compared to adequate functional health literacy [17]. There are a number of ways in which this relationship is supported and strengthened in the present study. First, there were consistent protective effects seen for both self-reported and objective measures of functional health literacy, Second, it is of longitudinal design and assesses incident foot ulceration as opposed to relying upon self-report history of ulceration as seen in previous studies. Overall, it provides compelling evidence that an important role of functional health literacy deficits in incident foot ulcer development is likely. Thus, it may be reasonable to attempt to mitigate potential effects, for example by taking “universal precautions”, that is, assuming all patients may have difficulties comprehending health information and minimising the risk of miscommunication through simplifying communication and confirming comprehension [35].

The tenets of diabetes-related foot prevention are heavily reliant on an individual (or their carer) routinely undertaking actions to minimize risk of developing foot disease. Basic requirements include, but are not limited to adhering to recommended footwear, performing regular foot inspections (including daily self-monitoring of skin temperatures) and maintaining optimal foot and skin hygiene [6] in addition to the demands of optimal diabetes self-management. It is thus imperative for patients to be engaged with and prioritize their healthcare needs. In our study, the HLQ domain *actively managing my health* had the greatest protective effect on incident foot ulceration, with a 83% reduction in odds of first foot ulcer in the model with MOCA, and 77% reduction in the model without, independent of baseline risk of foot disease in our study. Better scores have also recently been shown to have the strongest protective effects amongst all nine HLQ domains for admission and mortality [36]. Clearly, there is growing evidence this is a clinically important aspect of health literacy with critical implications for health outcomes. Individuals scoring poorly in this domain take little ownership, fail to see health as a personal responsibility, and subsequently are not engaged in their healthcare [23]. They are passive receivers of healthcare, perceiving it as something “done to them” [23]. Addressing this deficit could help prevent index foot ulceration, with potential strategies including understanding ways to improve engagement and ownership of health through co-design of health services, emphasizing shared decision making and goal setting and calling on existing support networks when providing diabetes-related foot care education.

Based on the key aspects of health literacy that may impact on incident foot disease development identified in this study, we propose a range of strategies to address poor health literacy which could be implemented or tested. First, assume all patients may have difficulties comprehending health information and simplify communication and confirm comprehension to minimize the risk of miscommunication, otherwise known as taking “universal precautions” in health literacy [35]. This approach has been shown to improve medication adherence in vulnerable patients with rheumatoid arthritis [37], but research into diabetic foot prevention is still lacking. Therefore, as a priority, we recommend further research on interventions to improve health literacy, and the effect of this improvement on long term diabetes complications such as foot disease. There is recent evidence suggesting health literacy interventions may be effective in improving health literacy, as well as knowledge, self-efficacy, and more importantly behavioural change as part of wider contemporary health behaviour models but this is limited by being at high risk of bias, and criticized for insufficiently poor reporting to allow for replication of interventions [38]. Furthermore, no studies have addressed diabetes-related foot disease or measured long-term disease outcomes. Third, attempt to improve patient engagement by using co-design to improve healthcare service delivery, as well as shared decision making and goal setting at the individual level. Finally, engage an individual’s support networks and community to attenuate the effects of poor health literacy. Support networks play an important role in diabetes management and complications prevention [39], and having poor informational, emotional and practical supports have been associated with higher rates of macrovascular complications in diabetes [40].

Cognitive decline is a crucial issue afflicting people with diabetes with significant implications. People with diabetes are at a 50% increased risk of having two or more cognitive deficits that interfere with daily activities [41], and cognitive impairment predisposes people with diabetes to acute complications such as severe hypo or hyperglycaemia [42]. Crucially, poorer cognition is associated with misunderstanding of the onset or etiology of ulceration amongst people attending a diabetes-related foot unit [43], although the role of cognitive impairment in re-ulceration has previously been dismissed [44]. Cognitive skills are intrinsic to health literacy if defined as an individual skillset or deficit as we have in this study. Thus we specifically assessed the potential confounding effect of cognitive impairment with the MOCA on associations of health literacy with foot ulceration, as it is important to consider in clinical practice in tandem with health literacy. In our modeling, adjusting for cognitive impairment with the MOCA strengthened the association of the domain *actively managing my health* with foot ulceration, but weakened the associations of functional health literacy and HLQ domain 9 (*understanding health information well enough to know what to do*) to the extent that these were no longer statistically significant. The logical explanation is that functional health literacy assessed by both domains (i.e. basic reading and writing of health information) is most dependent on cognitive skills. We hypothesize that the HLQ domains not affected by confounding by the MOCA score may indeed reflect compensation for these cognitive deficits as previously described in the literature [45]. Indeed, recent trends in health literacy research have identified health literacy as not just a skill or deficit borne by the individual, but a shared resource within a social network and the community to which one belongs [45]. A “health-literacy aware” community can attenuate effects of an individuals’ functional health literacy or cognitive deficits by providing a supportive environment to accommodate and facilitate the individual in managing their own health [45]. It becomes imperative to recognize that people with low functional health literacy may have significant cognitive deficits and vice versa, and subsequently to identify these subgroups as target populations for strategies to mitigate these factors when preventing diabetes-related foot disease. As part of patient-centered and biopsychosocial approaches to clinical care, such strategies may include providing simplified educational interventions, providing more frequent reviews, and engaging with an individuals’ support network or carers to undertake preventative care.

The key strength of our study is that we investigated development of an index foot ulcer, rather than recurrent foot ulcers, which has been highlighted as a key gap in diabetes-related foot ulcer prevention research [6, 7]. Another is that we measured a wide range of potential confounders including cognitive impairment which enabled us to assess its impacts on relationship between health literacy and foot ulcer development. Our study also had a low attrition rate. Furthermore, by using a range of health literacy measures, we were able to demonstrate key differences between the domains and the importance of a holistic health literacy assessment beyond functional health literacy alone. However, findings from this study should be taken in context; participants were recruited from a single tertiary hospital in Hobart, which may potentially limit its generalizability to the wider community of people with diabetes. There was a competing risk for mortality in this study, but only a relatively small number of participants (sixteen) died before the end of follow-up without an incident ulcer occurring, and we used inverse probability weighting to reduce the risk of bias from loss to follow up. We further acknowledge that in 2019 the International Working Group for the Diabetic Foot (IWGDF) updated foot risk classification tiers [6] some years after the study protocol was written and baseline data collected, however in all our models the effect of health literacy and cognitive impairment on foot ulcer development was not affected by baseline risk and our findings are unlikely to be significantly impacted by this change. As participants did not receive feedback of their sTOFHLA or HLQ scores, we were unable to assess whether such feedback would have any psychosocial impacts on participants.

To conclude, this is the first study to identify inadequate health literacy and cognitive impairment as important risk factors for incident foot disease development. Better implementation of current strategies and trials to test new interventions to address the impacts of these deficits on diabetes-related foot disease is essential.

## Supplementary Information

**Additional file 1: Supplementary Table S1.** Baseline characteristics of participants who did and did not complete the study.

## Data Availability

Some or all datasets generated during and/or analyzed during the current study are not publicly available (due to restrictions that their information could compromise the privacy of research participants) but are available from the corresponding author on reasonable request.

## Abbreviations

SHELLED study: Southern Tasmanian Health Literacy and Foot Ulcer Development in Diabetes study
S-TOFHLA: Short form Test of Functional Health Literacy in Adults
HLQ: Health Literacy Questionnaire
PHQ-9: Patient Health Questionnaire
MOCA: Montreal Cognitive Assessment
