# Supplementary material for: Health Literacy predicts incident foot ulcers after 4 years – the SHELLED cohort study
Source: J Foot Ankle Res. 2023 Jul 27;16:45. doi: 10.1186/s13047-023-00644-w (PMC10373326; doi:10.1186/s13047-023-00644-w)
Supplement: Supplementary file 1 — Additional file 1: Supplementary Table S1. Baseline characteristics of participants who did and did not complete the study. [file 13047_2023_644_MOESM1_ESM.docx]

Supplementary Table S1: Baseline characteristics of participants who did and did not complete the study

| Variable | Completed study (n=191) | did not complete study (n=31) |
| --- | --- | --- |
|  |  |  |
| Age | 60.6 (10.3) | 65.9 (12.2) |
| Male, n (%) | 108 (56.5) | 22 (71.0) |
| Years of formal education | 11.4 (3.4) | 10.4 (2.2) |
| STOFHLA score (0-36) | 32.5 (5.8) | 27.8 (10.4) |
| HLQ Domain Scores |  |  |
| 1: Feeling understood and supported by health professionals | 3.25 (0.53) | 3.31 (0.45) |
| 2: Having sufficient information to manage health | 3.06 (0.45) | 3.06 (0.49) |
| 3. Actively managing my health | 2.80 (0.51) | 2.88 (0.45) |
| 4. Social support for health | 2.95 (0.61) | 3.08 (0.53) |
| 5. Appraisal of health information | 2.83 (0.57) | 2.81 (0.57) |
| 6. Ability to actively engage with health professionals | 4.05 (0.70) | 4.14 (0.60) |
| 7. Navigating the healthcare system | 3.93 (0.64) | 3.99 (0.67) |
| 8. Ability to find good health information | 3.84 (0.77) | 3.84 (0.69) |
| 9. Understanding health information well enough to know what to do | 4.00 (0.69) | 3.99 (0.67) |
| BMI (kg/m^2^) | 33.8 (8.3) | 32.1 (6.7) |
| Duration of diabetes (years) | 17.2 (13.4) | 22.5 (13.0) |
| Insulin therapy, n(%) | 147 (77.0) | 26 (83.9) |
| PHQ-9 (0-27) | 7.3 (6.4) | 7.1 (5.6) |
| Diabetes Distress (0-6) | 1.8 (0.8) | 1.6 (0.7) |
| DMSES (0-10) | 9.5 (1.8) | 9.6 (1.5) |
| MOCA (0-30) | 25.9 (3.4) | 24.4 (3.9) |
| Diabetes Knowledge (0-100) | 74.0 (18.7) | 70.4 (20.5) |
| Current smoker, n(%) | 30 (15.7) | 3 (9.7) |

Data presented as mean(SD) unless otherwise indicated. Numbers in brackets for each variable represent possible score range

S-TOFHLA: short form Test of Functional Health Literacy in Adults; HLQ: Health Literacy Questionnaire; BMI: Body Mass Index; PHQ-9: Patient Health Questionnaire – 9 items; DMSES: Diabetes Management Self-Efficacy Scale; MOCA: Montreal Cognitive Assessment; HLQ domains 1-5 are scored out of 4, and domains 6-9 are scored out of 5.
